# Colonization effect of *Beauveria bassiana* (Bals.) Vuill. on tomato plant and *Bemisia tabaci*

**DOI:** 10.1038/s41598-025-00562-w

**Published:** 2025-05-16

**Authors:** Entesar Nahed Haron, Dalia Mohamed Ahmed Hassan, Eman El-Said, Nehal A. Zaid, Sahar F. Deraz, Ehab A. Serour

**Affiliations:** 1 Piercing and Sucking Insects Department, Plant Protection Research Institute, Agricultural Research Center, Giza, Egypt; 2 Acarology Department, Plant Protection Research Institute, Agriculture Research Center, Giza, Egypt; 3 General Entomology, Faculty of Science, Al-Azhar University (Girl’s Branch), Cairo, Egypt; 4 Protein Research Department, Genetic Engineering and Biotechnology Research Institute (GEBRI), City of Scientific Research and Technological Applications (SRTA-City), New Borg Al-Arab City, 21934 Alexandria Egypt

**Keywords:** Entomopathogenic fungi, Whiteflies, Tomatoes, Defense mechanisms, Tannins, Flavonoids, Growth promotion, Endophytes, Microbiology, Plant sciences

## Abstract

Whitefly (*Bemisia tabaci*) is an insect threatening tomato production in Egypt. This study investigated the impacts of the entomopathogenic fungi species, *Beauveria bassiana*, isolate against *B. tabaci* on tomato plants under natural conditions in two seasons (2023–2024). Conidial powder was directly applied to the soil. Fungus was added to the fertilization treatments (BF) and was compared with fertilization (CF) and control (C0). The findings indicated notable significant variations in the population densities of *B. tabaci* in comparison to the other groups in two seasons. This fungus can also be used as a growth enhancer besides being a biopesticide for tomato crops. Tomato leaf samples were collected in three growth phases: vegetative, flowering, and fruiting phases in addition to ripe tomato fruits. Collected leaves were dried and used to detect defense mechanisms through estimating phenolic compounds such as tannins and flavonoids and total protein content, while tomato fruits were used to estimate ascorbic acid level as a growth promotion indicator in the tested tomato plants. *B. bassiana* -treated plants showed a significant increase in total tannins compared to fertilization-treated plants and non-significant increase compared to control. While Total Protein Content (TPC) was significantly higher in fertilization-treated plants than in *B. bassiana* -treated plants and control it was only increased significantly in the bioagent treatment than in the control. For total flavonoids, a non-significant increase was detected in total flavonoids content in *B. bassiana*-treated plants than in fertilization- treated plants and controls. *Beauveria bassiana* -treated tomato fruits recorded the highest value of ascorbic acid content, which significantly increased than fertilization treatment and non-significantly increased compared to the control. Generally, the interaction between treatments and growth phases in total tannin content, total protein content, and total flavonoid content was not statistically significant, which means there is no behavior for *B. bassiana* treatment on the plant resistance mechanism during the different growth stages, and the highest level for each was recorded in the flowering phase compared to the vegetative and fruiting phases. Also, the findings indicated the highest yield was represented by adding *B. bassiana* to the soil. The obtained results from this study refer to the beneficial role of *B. bassiana* in systemic resistance induction stimulated by tannin content in the tested tomato plants against whitefly attacks.

## Introduction

Tomato is an important horticultural plant with a high level of bioactive compounds. These compounds are important as health-affecting compounds, especially for preventing chronic diseases and improving health^1^. However, challenges such as insect pests and diseases can lead to significant losses in both the quality and yield of tomato crops^2^. Whitefly *Bemisia tabaci* (Gennadius) (Hemiptera: Aleyrodidae) is an insect threatening tomato production^3^. Whitefly produces honeydew which stimulates the infection of sooty mold on the plant parts. This black sooty mold interferes with the photosynthesis process in the plant, therefore reducing production and product quality^4^. Additionally, whiteflies vector about 350 pathogenic plant viruses, which cause economically destructive diseases in vegetables and other crops^5,6^. Therefore, controlling this sucking pest insect is an important target in Egypt; regular use of synthetic pesticides causes residue problems and resistance/insect tolerance; therefore, it requires an alternative for managing pests and diseases^7,8^.

Entomopathogenic fungi (EPF) were proved to be good natural enemies of herbivores used in biological control purposes, with high commercialization potential^9,10^; some of the entomopathogens were reviewed to be promising candidates for substituting chemical insecticides^11^, such as *Beauveria* sp. and *Metarhizium* sp., which have been reported to act as pest -controlling factors in addition to working as growth promoters^12–14^. Soil, which is the main EPF reservoir, has been deemed the most suitable habitat for the application of these biocontrol agents^15,16^ in the form of biofertilizers^17^. Beneficial microorganisms, such as fungi, viruses, and bacteria, are essential to agriculture, serving as biopesticides and biofertilizers. These microorganisms contribute to soil fertility, support the growth of plants, and offer defense against pests and diseases, which ultimately decreases the dependence on chemical pesticides and fertilizers. The use of these biological agents not only improves crop production but also contributes to environmental conservation and food safety. Encouraging farmers to adopt these practices can lead to more sustainable and eco-friendlier agricultural techniques^18^. *Beauveria bassiana* (Ascomycota: Hypocreales) has been reported previously to be an effective mycoinsecticide for whitefly management^11,19^. This fungus can also be used as a growth enhancer besides being a biopesticide for tomato crops^20^. EPF can additionally function as a plant growth promoter and nutritional enhancer^21,22^. They have the remarkable ability to colonize a diverse array of plants, thereby exerting a substantial influence on the plant’s overall performance. EPF were experimentally introduced into the plants as endophytes to combat pests under natural conditions^23^.

Numerous recognized defense mechanisms have developed to limit the impact of insect attacks^24^. These mechanisms primarily consist of antifeedant or toxic substances that restrict insect activity^25^. Moreover, *B. bassiana* has been confirmed to reduce herbivory once it colonizes plants as endophytes^26^. The specific mechanism through which *B. bassiana* activates defense mechanisms in tomato plants against pests is still not understood^11^. On the other hand, the ecological relationship between plant tissues and insects is complicated^27^, as chemical interactions inducing plant responses may target insect resistance in several ways, like the accumulation of defensive compounds that affect reproductive success, feeding efficiency, and plant selection by the pest^28^. For example^29^, reported that cassava resistance to whiteflies was based on an antibiosis mechanism due to leaf content of phenolic compounds, free sugars^30^, alkaloids, tannins, flavonoids^31^, and proteins^32^. Flavonoids and tannins, classified as phenolic compounds, have been identified as defensive agents in plants. These compounds protect the plant against the whitefly pest by influencing the development, behavior, and growth of the pest^33^ through decreasing the nutrient content of the plant host sections to the insect and consequently repelling feeding by *B. tabaci*^34^. According to^35^ an inverse relationship was found between *B. tabaci* plant population with tannins and flavonols, which shows that increasing the efficacy of these compounds could play a role in the biocontrol of whiteflies in host plants. Phenolics are some of the secondary metabolites found in plants; they are produced via the malonic acid and shikimic acid pathways through phenylpropanoid metabolism^36^.

Vitamin C (ascorbic acid) is an essential element in a person’s daily diet as it boosts the health of humans. It helps to obviate iron deficiency, reinforces the immune system and decreases the risk of heart diseases^37^. Since the humans are not capable of synthesizing vitamin C from fructose like plants do^38^, they depend totally on the external sources of vitamin C^39^. The most known sources of vitamin C are fruits (citrus fruits), in addition to vegetables, such as tomatoes and bell peppers^37,40^.

In this study, we attempt to address a research gap by exploring the use of B. bassiana fungi directly in soil as a novel approach to controlling B. tabaci in tomato crops under natural field conditions. While most earlier studies have focused extensively on foliar sprays or seed treatments, our research investigates the potential of soil inoculation to enhance plant resistance and contribute to sustainable pest management. This approach offers new insights into the role of soil-applied entomopathogenic fungi in integrated pest management strategies.

## Methods

### Fungal culture

The bioagent *Beauveria bassiana* was originally isolated from soil^41^. Conidia of entomopathogenic fungi were used. Boiled rice was autoclaved in Erlenmeyer flasks for 20 min at 121 °C. The separation of rice grain coagulation was achieved by vigorously shaking flasks. Subsequently, the flasks were allowed to cool down to room temperature and inoculated with 1 ml of conidia suspension containing 10^8^ conidia/ml. The flasks were then incubated in the dark at a temperature of 25 ± 1 °C for 2–3 weeks. Finally, the sporulated rice was dried and grounded by using the desiccant technique described by^42^: an amount of sodium hydroxide, serving as a desiccant. Each 200 gm of dried rice had 10^8^ spores/5 L of water.

### Field experiment

The investigation was conducted in the Experimental Station (Qaha) of the Plant Protection Research Institute (PPRI), Qalyoubia Governorate, Agricultural Research Center (ARC), Egypt, over two successive seasons (2023 and 2024). One variety of tomato (442) was used, and cultivation of seedlings of tomatoes was in September. The study included one fungus (*B. bassiana*) (B) and two control treatments, CF and C0 (untreated control). Untreated control (C0) was included to compare the effect of bioagent treatment with recommended fertilizers without any modifications. The *B. bassiana* was applied directly to the soil as a conidial powder amendment, which was applied three timeline synchronization with fertilization schedules. Treatments were arranged in a randomized complete block design:

- (BF) bioagent (200gm/ row) + fertilizers.
- (CF) fertilizers were repeated according to the Egyptian Ministry of Agriculture recommendation.
- (C0) Control (soil wasn’t any treatment).

A one-meter-wide separation was maintained between the blocks. Each block consisted of five rows, with ten plants in each row, arranged according to a Randomized Complete Block Design. The normal agricultural practices were applied. Treatments were repeated three times in the season. All examined plants were exposed to the natural infection of whiteflies. A random sample of ten leaves was collected per treatment each week during the early morning hours. To ensure representative sampling, the leaves were taken from multiple plants; sampling leaves were put in labeled paper bags and then transported to the laboratory to examine the total number. of *Bemisia tabaci* nymphs (all stages of nymph) by using a binocular microscope. Observations continued for nine weeks; it corresponded to the timing of fertilizer applications. The observation schedule was designed to assess the cumulative effects of *B. bassiana* treatment on *B. tabaci*. The efficiency of the experiment was based on the reduction percentage of whiteflies’ nymphs’ population according to the formula of^43^ as follows:

$${\text{Reduction}}\;{\text{percentage}} = \left\{ {1 - \left( {{\text{C}}1 \times {\text{T}}2/{\text{C}}2 \times {\text{T}}1} \right)} \right\} \times 100$$

where C1 = population in control before application. (CF or C0).

C2 = population in control after application. (CF or C0).

T1 = population in treatment (BF) before application.

T2 = population in treatment (BF) after application.

### Assessment of tomato fruit yield

Tomato yield is measured by weighing only high-quality tomatoes weekly as much as six harvesting times, at 12 to 17 weeks after planting.

### Chemical analysis

#### Sampling

Tomato leaves were sampled on the three growing phases (vegetative, flowering, and fruiting phases) around the season, in addition to tomato fruit sampling. The collected leaf samples were dried; then the fresh and dried plant leaf materials served as a source of extraction of proteins and secondary plant metabolites, while tomato fruits were used to estimate vitamin C levels.

### Defense mechanisms indicators analyses

Evaluation of defense mechanisms was conducted by performing some analysis of total proteins and the two secondary metabolites, tannins and flavonoids, that are previously reported to be effective against whiteflies invading.

#### Total protein content

Total protein content was estimated in tomato leaves by the^44^.

#### Total tannins

Total tannin content was analyzed according to^45^.

#### Ascorbic acid levels

Determination of Vitamin C Concentration was performed on the mature tomato fruits by titration method as mentioned by the University of Canterbury’s website (www.outreach.canterbury.ac.nz) as follows:

Tomato fruits were blended, and the juice was strained through cheesecloth to remove pulp and seeds.

Twenty mL of the sample solution was pipetted into a 250 mL flask. The sample was titrated with 0.005 mol/L iodine solution. Before the endpoint, the original color of the sample solution is retained as it is, and when the ascorbic acid has totally been oxidized, a slight excess of added iodine forms a dark color (this is the titration endpoint). The volume of iodine solution used was calculated.

The number of moles of ascorbic acid reacting was calculated by using the following equation of the titration.

$${\text{ascorbic acid}} + {\text{I}}_{{2}} \to {\text{ 2 I}}^{ - } + {\text{dehydroascorbic acid}}.$$

The concentration was evaluated in mg of ascorbic acid/100 mL of tomato juice.

### Statistical analysis

All statistical analyses were performed in SPSS (version 16.0 for Windows, USA). Data were subjected to ANOVA to assess the effects of *B. bassiana* on total counts of nymphs per plant leaf. Comparisons of means were performed using Duncan’s multiple range test (= 0.05). All results of the reduction percentage of whiteflies are expressed as mean ± SE. Statistics were considered significant if the *P* value was < 0.05.

## Results

### Season 2023

**Fig. 1:**
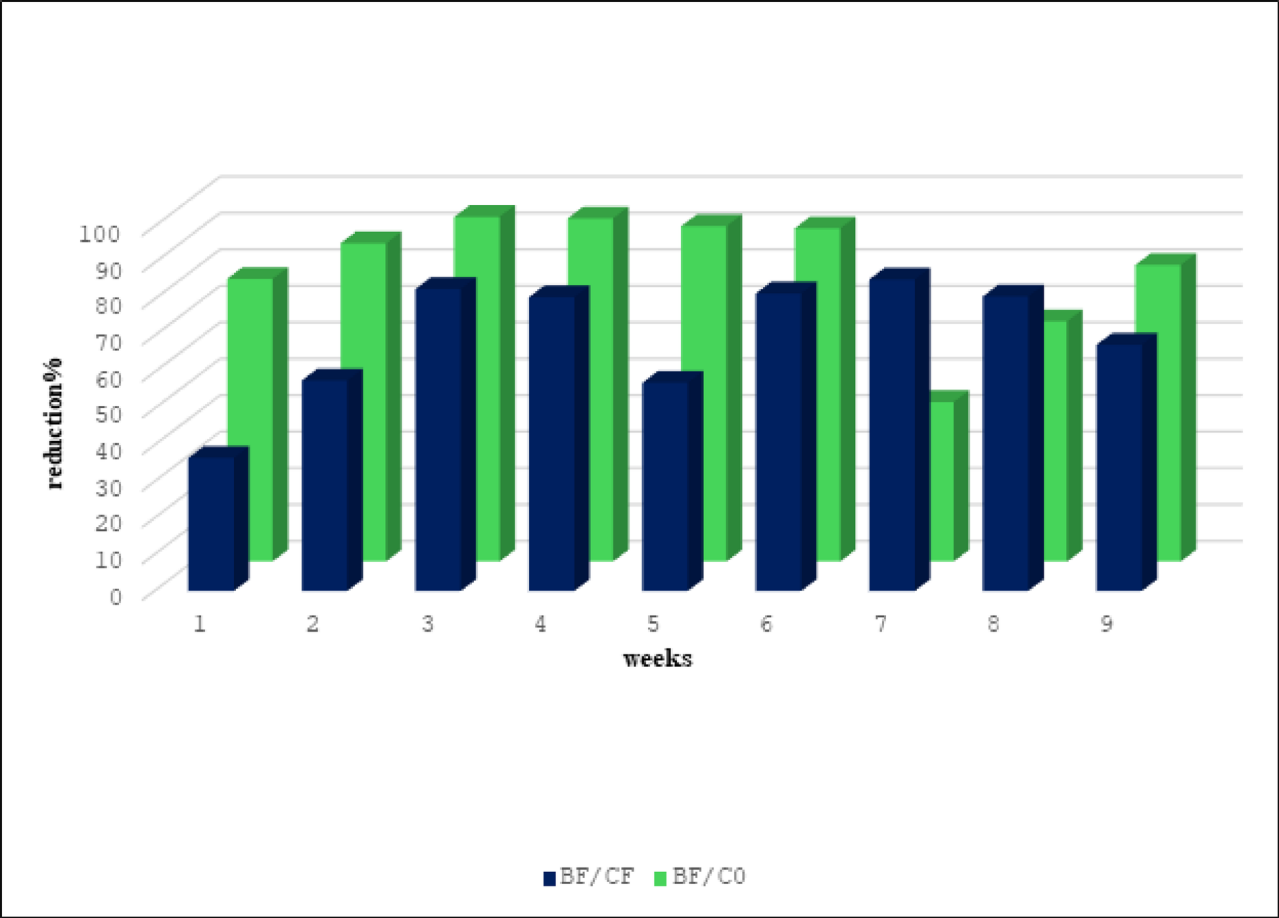
Reduction% of *B. tabaci* nymphs on a tomato plant after application during season 2023.

Season 2023 in the results revealed that there were differences in population densities of whiteflies for BF, CF, and C0. Significant difference in the mean number of pests after introducing fungi into the soil with fertilizers when compared with CF or C0 (Table 1). In the first week after application, the means were 61.2, 73, and 86.2 for BF, CF, and C0, respectively. While, in the ninth week, the means were 1.8, 4.2, and 4.4 for BF, CF, and C0, respectively. The results, shown in Fig. 1, showed that there was a reduction% when adding fungus, whether when compared to the CF or C0. In the first week, the reduction % was 36.49 and reached 67.53 in the last week when compared BF with CF. While it was 77.56 in the first week, it reached 81.34 in the last week when comparing BF with C0.

**Table 1:** Mean ± S. E of immature whitefly on tomato plant after different intervals post-application during season 2023.

Treatments	First application			Second application			Third application		
	First week	Second week	Third week	Fourth week	Fifth week	Sixth week	Seventh week	Eighth week	Ninth week
BF	61.2 ± 13b	45.2 ± 9.47b	22.6 ± 2.37c	6.2 ± 0.9c	18 ± 2.66b	29.8 ± 5.27c	7.4 ± 0.81b	11.2 ± 1.4b	1.8 ± 0.37b
CF	73 ± 5.5b	81 ± 6.78b	99.8 ± 24.14b	24.2 ± 5.8b	31.8 ± 2.7b	122.8 ± 12.2b	38.6 ± 3.9a	44.2 ± 11.47a	4.2 ± 0.86a
C0	86.2 ± 5.4a	162.4 ± 16.47a	189.4 ± 21.9a	48.4 ± 4.06a	102.8 ± 7.05a	157.8 ± 12.11a	6 ± 1.41b	15 ± 3.18b	4.4 ± 0.92a
F	15.01**	26.59**	19.59**	26.36**	96.77**	40.59**	56.86**	6.69**	3.61*
Sig	0.00	0.00	0.00	0.00	0.00	0.00	0.00	0.01	0.06

*BF* bioagent + fertilizers, *CF* fertilizers, *C0* Control.

Means in a Column followed with the same letter(s) are not significantly different at 5% level of probability. ** = Highly significant; * = significant.

### Season 2024

**Fig. 2:**
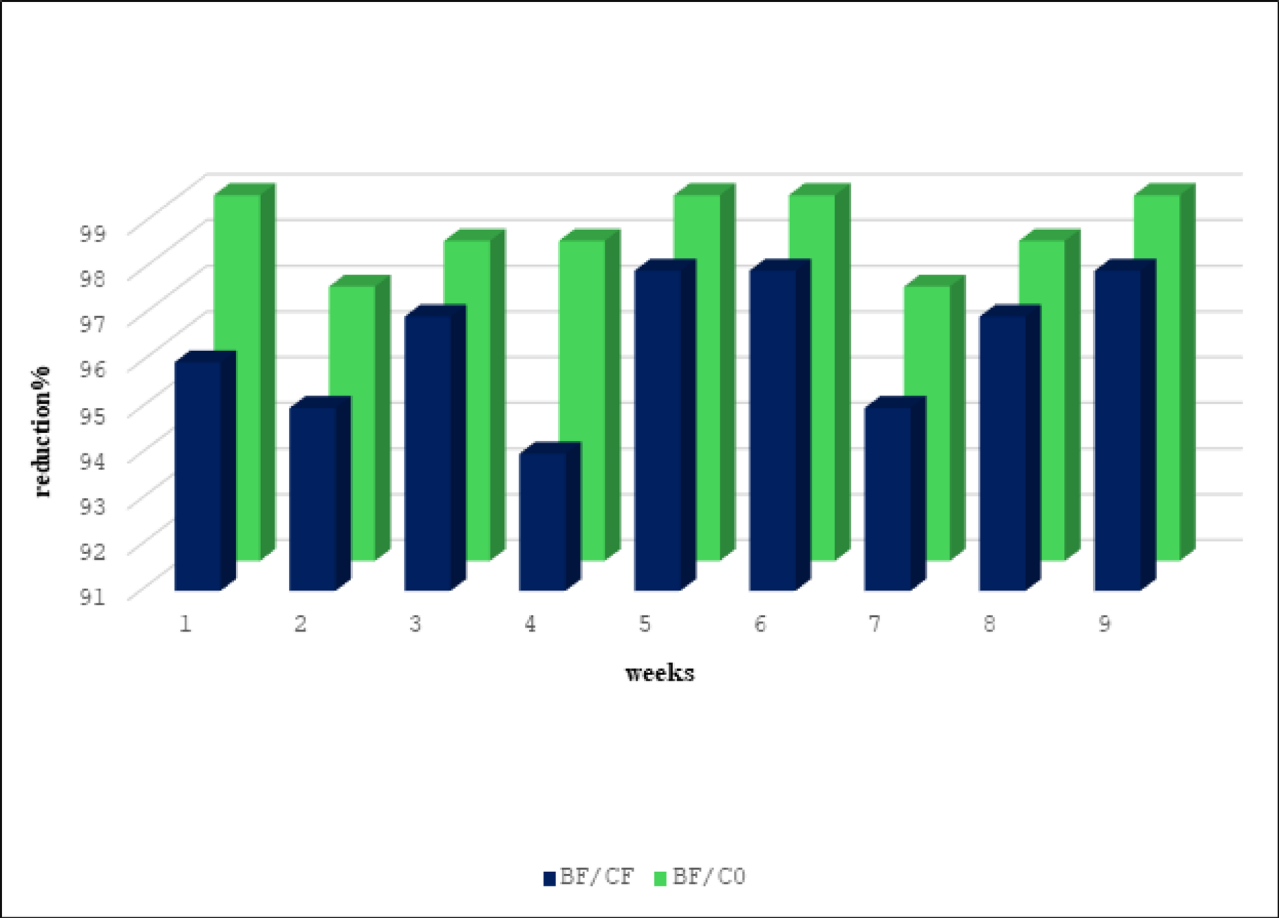
% reduction of *B. tabaci* nymphs on a tomato plant after application during season 2024.

Season 2024 in the results also revealed that there were differences in population densities of whiteflies for BF, CF, and C0. Significant difference in the mean number of recorded pest infestations after introducing *Beauveria bassiana* into the soil with fertilizers when compared with CF or C0 (Table 2). In the first week after application, the means were 3.4, 9, and 14.2 for BF, CF, and C0, respectively. While, in the ninth week after application, the means were 2.8, 13, and 11.2 for BF, CF, and C0, respectively. The results, shown in Fig. 2, showed that there was a reduction when adding fungus, whether when compared to the CF or C0. The reduction percentage was 96 in the first week, while it reached 98 in the 9th week when compared BF with CF. While it was 99 in the first week and the last week when comparing BF with C0.

**Table 2:** Mean ± S. E of immature Whitefly on Tomato plant after different intervals post-application during season 2024.

Treatments	First application			Second application			Third application		
	First week	Second week	Third week	Fourth week	Fifth week	Sixth week	Seventh week	Eighth week	Ninth week
BF	3.4 ± 1.2c	0.6 ± 0.4a	2.6 ± 1.3a	13.4 ± 1.03b	16 ± 4.27b	6.2 ± 1.11b	11.6 ± 4.18a	6.2 ± 1.4b	2.8 ± 1.01b
CF	9 ± 1.76b	1.2 ± 0.38a	7.8 ± 0.37a	22 ± 3.5a	79.2 ± 10.19a	26.6 ± 2.48a	22.4 ± 5.55a	17.7 ± 2.57a	13 ± 1.73a
C0	14.2 ± 1.77a	0.8 ± 0.38a	5 ± 1.41ab	24 ± 2.59a	65.4 ± 12.25a	31.4 ± 5.34a	17 ± 4.52a	14.1 ± 2.85a	11.2 ± 1.36a
F	11.08**	0.67ns	5.35*	4.79*	12.17**	14.94**	1.27ns	6.23*	15.12**
Sig	0.002	0.55	0.02	0.03	0.001	0.001	0.32	0.01	0.001

*BF* bioagent + fertilizers, *CF* fertilizers, *C0* Control.

Means in a Column followed with the same letter(s) are not significantly different at 5% level of probability.

** = Highly significant; * = significant; ns = not significantly.

### Tomato fruit yield

In 2023, Fig. 3 showed tomato yields were 500, 340, and 224 kg for BF, CF, and C0, respectively. The highest yield was represented by adding *B. bassiana* to the soil. Also, there were noticeable significant differences in yield (F = 19.87 and *p* < 0.05) when comparing the yield for each treatment (Table 3). The same observations were obtained in 2024; Fig. 4 showed tomato yields were 1322,713 and 461 kg for BF, CF, and C0, respectively. Also, there are noticeable significant differences when comparing the yield (F = 4.87 and *p* < 0.05) for each treatment (Table 3).

**Fig. 3:**
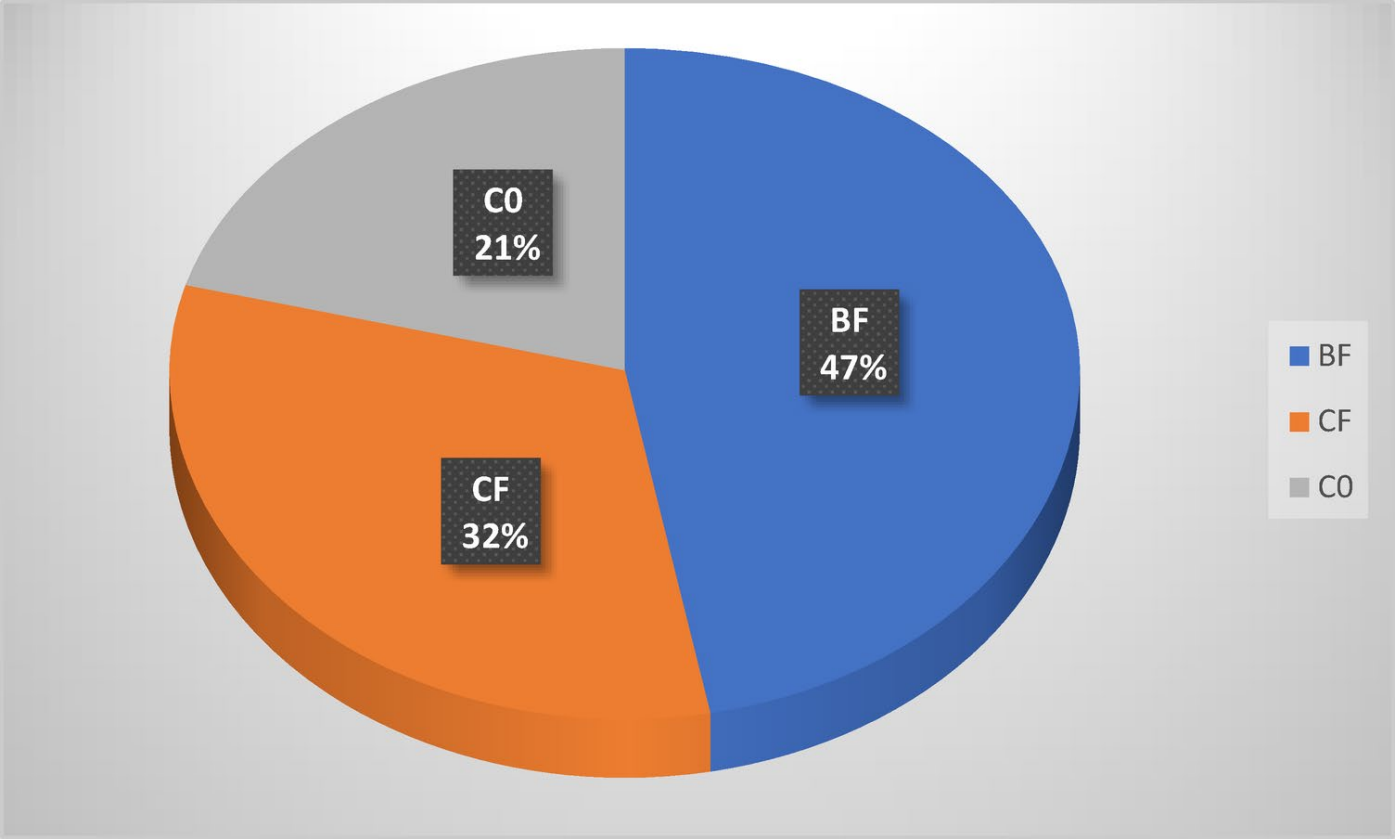
Yield (Kg/block) of tomato plants at season 2023.

**Table 3:** yield of tomato plant ± S. E season 2023 and 2024.

Treatments	Yield mean ± S. E 2023	Yield mean ± S. E 2024
BF	83.33 ± 3.3a	220.33 ± 48.95a
CF	56.66 ± 8.02b	118.83 ± 27.23ab
C0	37.33 ± 2.2c	76.83 ± 18.43b
F	19.87**	4.88*
Sig	0.00	0.00

Means in a Column followed with the same letter(s) are not significantly different at 5% level of probability.

** = Highly significant, * = significant.

**Fig. 4:**
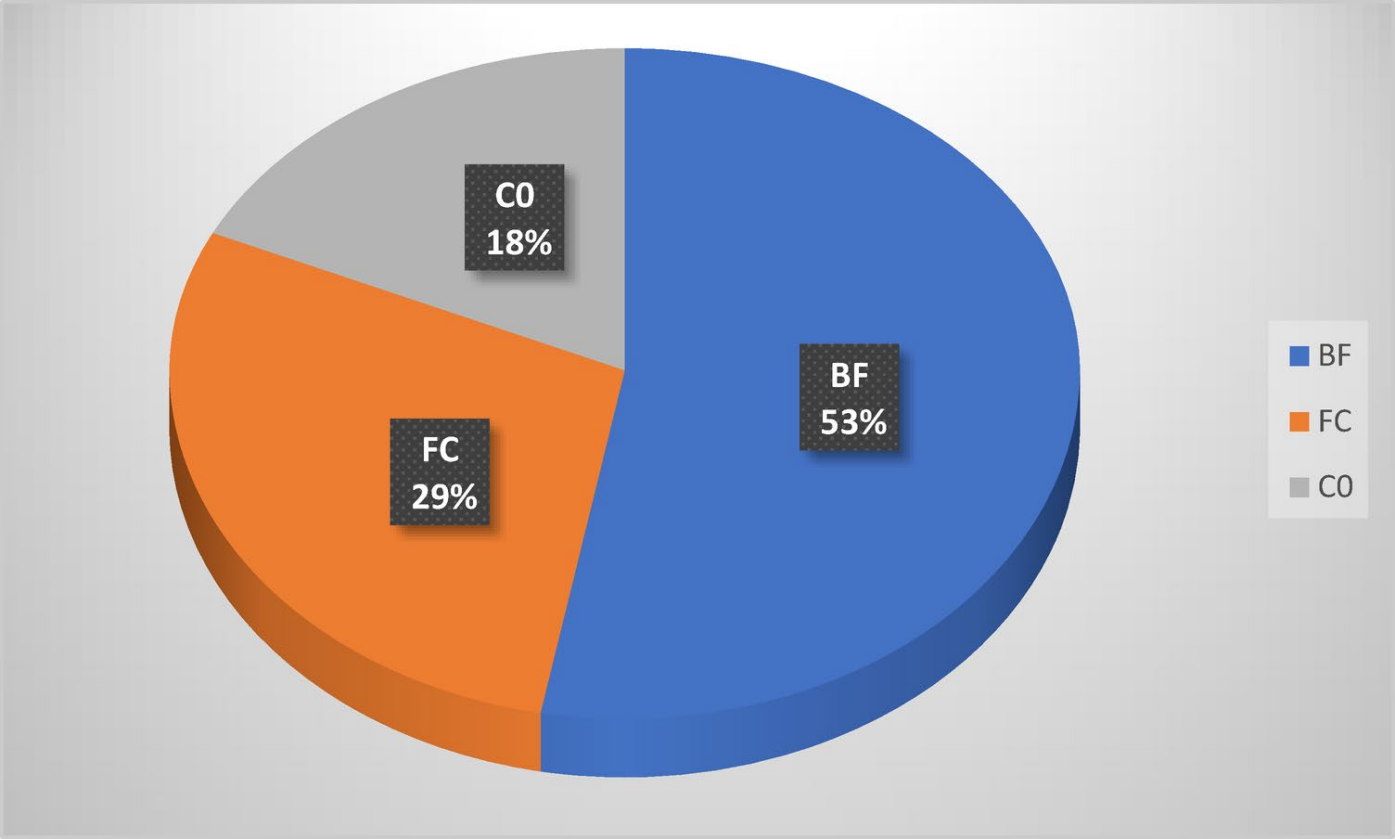
Yield (Kg/block) of tomato plant at season 2024.

### Chemical analysis

#### Total protein content (TPC) in the tested tomato plants

**Fig. 5:**
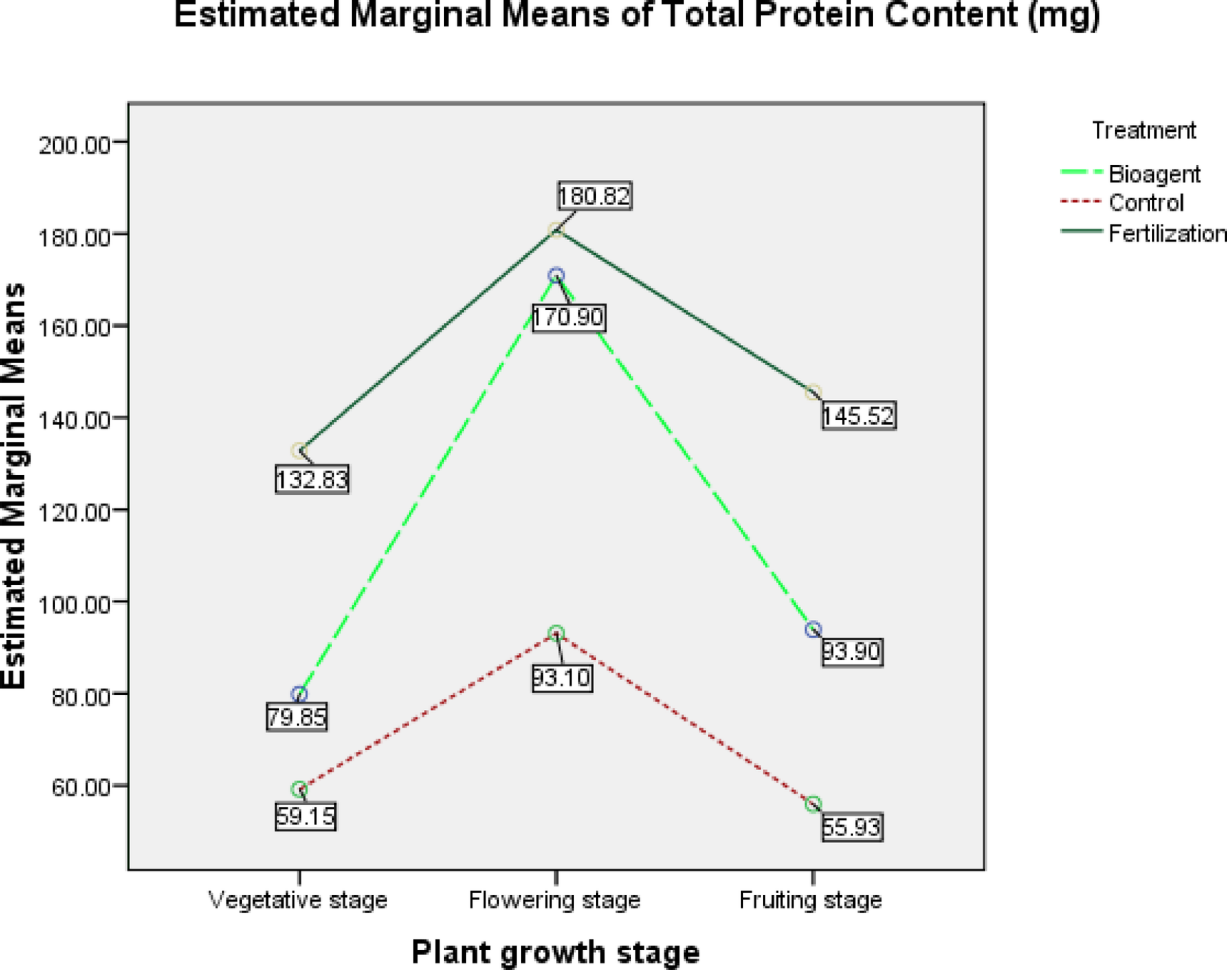
Estimated marginal means of total protein content (TPC) for treatments and growth phases.

A two-way ANOVA was conducted to examine a difference in total protein content among treatments and among growth phases of each treatment. The interaction between treatments and growth phases has no significant effect (*P* = 0.169) (Fig. 5). However, pairwise comparisons for either treatments or growth phases indicated that there is a significant effect in TPC between treatments, including control; plants treated with *B. bassiana* bioagent showed a significantly high content of total protein compared to control (MD = 45.491, P_0.05_ = 0.000), but plants treated with only fertilization showed the highest significant level of total protein compared to either bioagent treatment (MD = 38.174, P_0.05_ = 0.001) or control (MD = 83.666, P_0.05_ = 0.000) (Table 4) and (Fig. 5).

**Table 4:** Effect of *B. bassiana* treatment on Total Protein Content (mg/100 mg dry weight) in the tested tomato plants.

(I) Treatment	(J) Treatment	Mean difference (I–J)	Std. error	Sig.^a^	95% Confidence Interval for Difference^a^	
					Lower bound	Upper bound
Bioagent	Control	45.491*	9.675	0.000	25.165	65.817
	Fertilization	− 38.174*	9.675	0.001	− 58.501	− 17.848
Control	Bioagent	− 45.491*	9.675	0.000	− 65.817	− 25.165
	Fertilization	− 83.666*	9.675	0.000	− 103.992	− 63.339
Fertilization	Bioagent	38.174*	9.675	0.001	17.848	58.501
	Control	83.666*	9.675	0.000	63.339	103.992

Based on estimated marginal means (Fig. 5).

*The mean difference is significant at the 0.05 level.

The pairwise comparison for TPC between growth phases showed significant effect between each other; as TPC reached significantly its maximum level in the flowering phase compared to vegetative phase (MD = 57.662, P_0.05_ = 0.000) and fruiting phase (MD = 49.824, P_0.05_ = 0.000); while no significant difference between fruiting and vegetative phases in TPC (MD = 7.838, P_0.05_ = 0.428) (Table 5; Fig. 5).

**Table 5:** Total Protein Content (mg/100mg dry weight) in growth phases in the tested tomato plants:

(I) Plant growth phase	(J) Plant growth phase	Mean Difference (I–J)	Std. error	Sig.^a^	95% Confidence Interval for Difference^a^	
					Lower bound	Upper bound
Flowering phase	Fruiting phase	49.824*	9.675	0.000	29.498	70.151
	Vegetative phase	57.662*	9.675	0.000	37.336	77.988
Fruiting phase	Flowering phase	− 49.824*	9.675	0.000	− 70.151	− 29.498
	Vegetative phase	7.838	9.675	0.428	− 12.488	28.164
Vegetative phase	Flowering phase	− 57.662*	9.675	0.000	− 77.988	− 37.336
	Fruiting phase	− 7.838	9.675	0.428	− 28.164	12.488

Based on estimated marginal means (Fig. 5).

*The mean difference is significant at the 0.05 level.

#### Total tannins content (TTC) in the tested tomato plants

**Fig. 6:**
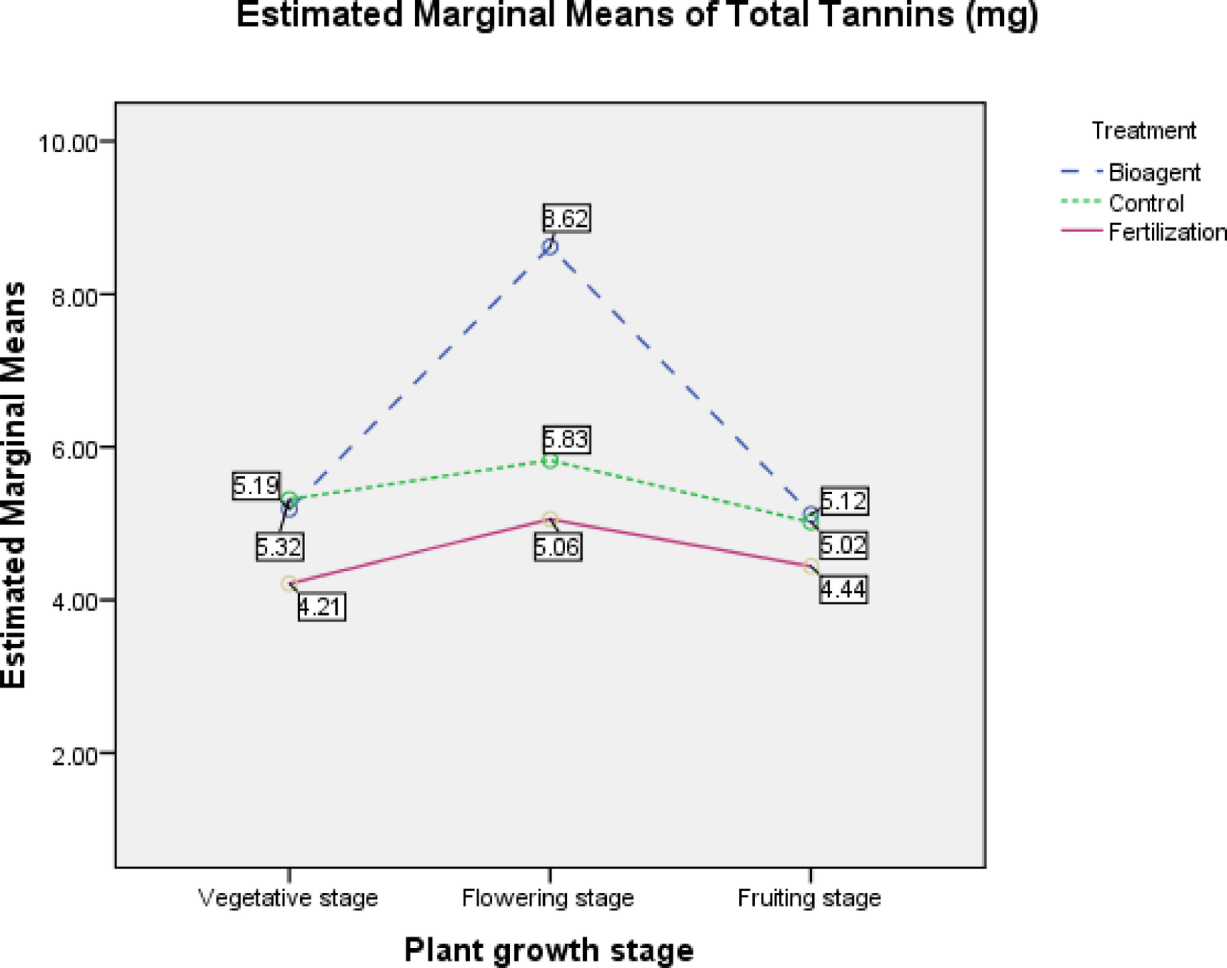
Estimated marginal means of total tannins content (TTC) for treatments and growth phases.

Total tannin content: collected data were statistically analyzed by two-way ANOVA, which was performed to examine a difference in total tannin content among treatments and among growth phases in the tested tomato plants. Statistical results showed that there is no significant interaction between treatments and growth phases (*P* = 0.233) (Fig. 6). But pairwise comparisons among treatments and among growth phases indicated that there is a significant effect in TTC between treatments compared to control; TTC in plants treated with *B. bassiana* bioagent increased significantly compared to the plants treated with only fertilization (MD = 1.739, P_0.05_ = 0.011), while no significant effect in TTC was found between *B. bassiana*- treated plants and control (MD = 0.922, P_0.05_ = 0.150). On the other hand, TTC in control was higher than that in only fertilization- treated plants (MD = 0.817, P_0.05_ = 0.200) (Table 6) and (Fig. 6).

**Table 6:** Effect of *B. bassiana* treatment on Total Tannins Content (mg/100 mg dry weight) in the tested tomato plants.

(I) Treatment	(J) Treatment	Mean difference (I–J)	Std. error	Sig.^a^	95% Confidence Interval for Difference^a^	
					Lower bound	Upper bound
Bioagent	Control	0.922	0.614	0.150	− 0.368	2.212
	Fertilization	1.739*	0.614	0.011	0.449	3.029
Control	Bioagent	− 0.922	0.614	0.150	− 2.212	0.368
	Fertilization	0.817	0.614	0.200	− 0.473	2.107
Fertilization	Bioagent	− 1.739*	0.614	0.011	− 3.029	− 0.449
	Control	− 0.817	0.614	0.200	− 2.107	0.473

Based on estimated marginal means (Fig. 6).

*The mean difference is significant at the 0.05 level.

The pairwise comparison for TTC between growth phases indicated that TTC significantly reached its highest level during the flowering phase compared to the vegetative phase (MD = 1.596, P_0.05_ = 0.018) and the fruiting phase (MD = 1.642, P_0.05_ = 0.015; but no significant difference between the fruiting and vegetative phases in TTC (MD = 0.047, P_0.05_ = 0.940) (Table 7; Fig. 6).

**Table 7:** Total tannins content (mg/100 mg dry weight) in growth phases in the tested tomato plants:

(I) Plant growth phase	(J) Plant growth phase	Mean difference (I–J)	Std. error	Sig.^a^	95% Confidence Interval for Difference^a^	
					Lower bound	Upper bound
Flowering phase	Fruiting phase	1.642*	0.614	0.015	0.352	2.932
	Vegetative phase	1.596*	0.614	0.018	0.306	2.885
Fruiting phase	Flowering phase	− 1.642*	0.614	0.015	− 2.932	− 0.352
	Vegetative phase	− 0.047	0.614	0.940	− 1.337	1.243
Vegetative phase	Flowering phase	− 1.596*	0.614	0.018	− 2.885	− 0.306
	Fruiting phase	0.047	0.614	0.940	− 1.243	1.337

Based on estimated marginal means (Fig. 6).

*The mean difference is significant at the 0.05 level.

#### Ascorbic acid (Vitamin C) levels in the tested tomato fruits

**Fig. 7:**
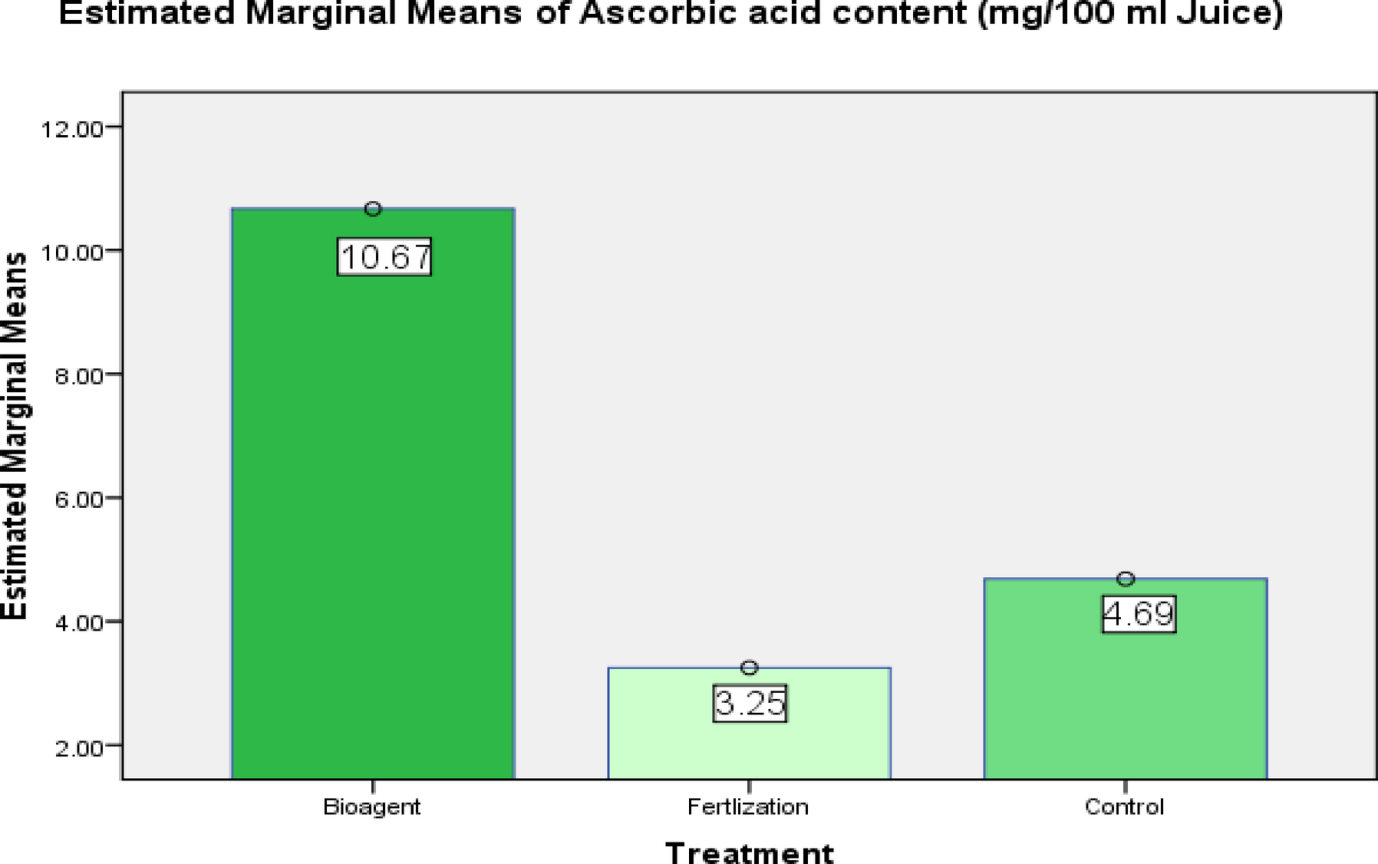
Estimated marginal means of ascorbic acid levels in the treated tomato fruits (*Bioagent is *B. bassiana*).

A one-way ANOVA was performed to determine the effect of the bioagent treatment on ascorbic acid levels, which were set as the dependent variable in the examined tomato fruits. The pairwise comparisons indicated that ascorbic acid level in the fruits was significantly increased by *B. Bassiana* treatment than which was in the fertilization only- treated plants (MD = 7.418, P_0.05_ = 0.028), however, this increase was non- significant compared to control (MD = 5.980, P_0.05_ = 0.064). Noteworthy, ascorbic acid levels were higher in the control than in the fertilization treatment, but no significant effect was found between them in the examined tomato fruits (MD = 1.438, P_0.05_ = 0.624) (Table 8; Fig. 7).

**Table 8:** Effect of *B. bassiana* treatment on ascorbic acid levels (mg /100 ml tomato juice) in the tested tomato fruits.

(I) Treatment	(J) Treatment	Mean Difference (I–J)	Std. error	Sigm^a^	95% Confidence Interval for difference^a^	
					Lower bound	Upper bound
Bioagent	Control	5.980	2.834	0.064	− .431	12.391
	Fertlization	7.418*	2.834	0.028	1.006	13.829
Control	Bioagent	− 5.980	2.834	0.064	− 12.391	0.431
	Fertlization	1.438	2.834	0.624	− 4.974	7.849
Fertlization	Bioagent	− 7.418*	2.834	0.028	− 13.829	− 1.006
	Control	− 1.438	2.834	0.624	− 7.849	4.974

Based on estimated marginal means (Fig. 7).

*The mean difference is significant at the 0.05 level.

## Discussion

Entomopathogenic fungi, *Beauveria bassiana* (Bals)Vuill. have proved significant efficacy as a biological agent for managing insect pests^46,47^. The present study evaluates the potential of *B. bassiana* that has colonized tomato plants as a regulator of *Bemisia tabaci* populations under field conditions. These results were like those obtained by^48,49^, which told that in addition to EPF’s well-known ability to regulate insect populations. Entomopathogenic fungi have also been found to colonize plants. A study conducted by^16^ examined the potential of two EPF strains, *Metarhizium brunneum* (Hypocreales: Clavicipitaceae) and *B. bassiana*, as regulators of *Philaenus spumarius* L. (Hemiptera: Aphrophoridae) *populations* in both direct and indirect control scenarios. The results revealed that the *M. brunneum* strain showed higher pathogenicity towards *P. spumarius* when applied directly and demonstrated more efficient colonization of the host plant *Sonchus oleraceus*. Also^23^, studied the effectiveness of entomopathogens as endophytes in sweet sorghum plants against *Sesamia nonagrioides* (Lepidoptera, Noctuidae) larvae that was examined under natural conditions. The introduction of these entomopathogens resulted in a reduction of infestation by 20-30% and a decrease in tunneling length by 19–43%. Additionally, there was a significant increase in larval mortality by 57–82%, while the growth of sorghum remained unaffected. Our findings also demonstrated that *B. bassiana* effectively managed whiteflies when incorporated into the soil alongside fertilizers. Our results were like those obtained by^50^, which demonstrated that a weekly application of *Aschersonia aleyrodis* at a concentration of 10^8^ conidia/mL led to a remarkable 90.6% decrease in whitefly populations. Furthermore, the tomato yield increased to an average of 1009 grams per plant, producing approximately 16 fruits per plant. The rates of mycosis and mummification were recorded at 96.6% and 97.3%, respectively, underscoring the fungus’s effectiveness as a biocontrol agent against *B. tabaci* in tomato cultivation. These outcomes align with those conducted by^21^, which summarized the ability of microorganisms to enhance crop plants’ defense against pathogens. Additionally, their study emphasized the potential of beneficial microorganisms as biofertilizers or biopesticides to support and enhance crop production while providing protection. Farmers depend on the correct choice of fertilizers and pesticides to enhance crop production and manage pests. The term “biofertilizer” refers to soil microorganisms that improve nutrient accessibility and absorption in plants while also promoting growth by managing pathogens. These biofertilizers can also promote plant growth by regulating plant pathogens^17,51^ conducted a study where they used three EPF isolates, *B. bassiana, M. brunneum*, and *Metarhizium robertsii* (Hypocreales: Clavicipitaceae), for seed inoculation of wheat and beans. They assessed the impact on the population growth of aphids, specifically *Rhopalosiphum padi* and *Aphis fabae* (Hemiptera: Aphididae). The results showed that inoculations with *M. robertsii* and *B. bassiana* led to a decrease in aphid populations compared to the control treatments. Surprisingly, *Metarhizium brunneum* (Hypocreales: Clavicipitaceae) had the opposite effect, increasing the populations of both aphid species. The study presented by^52,53^ highlights the potential of biopesticides and biofertilizers for use in both organic and conventional farming practices. According to^54^ endophytic fungi play a crucial role in stimulating the growth of host plants through the direct production of secondary metabolites. These metabolites enhance the plant’s ability to withstand both biotic and abiotic stresses. Moreover, these fungi have the capability to biosynthesize medically significant "phytochemicals," which were previously believed to be exclusively produced by the host plant. A research study by^55^ assessed the efficacy of the entomopathogenic fungi *Metarhizium anisopliae* and *B. bassiana* in managing the tomato leaf miner (*Tuta absoluta*). The results showed that these fungi notably reduced the distance larvae traveled from the egg to the entrance of their galleries, as well as their total weight. Furthermore, the fungi successfully colonized tomato plants, showing mycelial growth within 7 days and reaching full development by 12 days, under conditions of 25 ± 2 °C and 95% humidity.

The results obtained from our study have indicated that defense mechanisms have been induced by *B. bassiana* in the examined tomato plants against *B. tabaci*; this type of defense is named induced systemic resistance^56^. The study presented by^57^ stated that the root inoculation was performed using nine well-defined bacterial and fungal strains, in addition to two microbial consortia, on tomato plants cultivated under intensive agricultural conditions. The assessment concentrated on multiple aspects, such as plant development, fruit quality, production yield, and the presence of pests and diseases. Although the majority of microbial strains exhibited minimal impact, the fungal strains *Trichoderma afroharzianum* T22 and *Funneliformis mosseae* notably enhanced the yield of marketable tomatoes. Furthermore, the introduction of various fungal strains resulted in a significant decrease in the occurrence of the harmful leaf-mining pest *Tuta absoluta*, a phenomenon that was not seen with bacterial inoculants.

In the present study, total tannins showed a significant increase in *B. bassiana* -treated plants compared to only fertilization treatment and water control, which refers to the axial role of *B. bassiana* in improving tannin contents during whitefly attack. Noteworthy, tannin contents non-significantly increased in water control plants than in only- fertilization -treated plants; this may be according to the double induction by the biotic stress of whiteflies attack and the abiotic stress of lack of nutrients in the control plants. When it is induced, this phenolic compound accumulates in extending cells from the plant epidermis named the glandular trichomes^1,58^; these trichomes are known to accumulate, store, and release plant secondary metabolites to serve as plant self-defenses that are triggered upon biotic or abiotic stress in a plant and precipitated at the attacked site^19,58–61^.

The defense mechanism of tannins is usually based on their antifeedant action to whiteflies, as they reduce the nutritive availability through chelating of metal ions or nonspecific protein precipitation^34,45,62^.

Increasing the total protein content in bioagent- treated tomato plants compared to water control in the present study is an expected action based on previous studies; a strong relationship was found between phenolic accumulation and the activation of some defense-related enzymes such as β-1,3-glucanases, chitinases, polyphenol oxidases, and peroxidases, and also other key enzymes in the phenylpropanoid and isoflavonoid pathways, which play a powerful role in pest and pathogen attack resistance in plants^1^. For instance,^35,45,63^ reported that peroxidase activity was increased coincide with increased tannin in the cassava resistant genotypes and both were involved in increasing resistance to whiteflies; it was found that a significant negative correlation was observed between cassava leaf damage scores and peroxidase activity, and similarly between tannin and damage, which prove that tannin and peroxidase have an effective role together in cassava resistance to whiteflies; this effective role is owing to peroxidase activity in producing phenoxy and other oxidative radicals which act together with tannins to deter the feeding by whiteflies and/or produce toxins which reduce the plant digestibility and causes nutrient deficiency to whiteflies and consequently leads to drastic effects on the insect’s growth and development^45,64^.

Also, many studies reported previously that flavonoids are toxic to herbivore insects and consequently protect plants against invading, as they are excreted as a response to plant invading by insect pests or herbivores^1,65,66^. Moreover, the oxidation of flavonoids by oxidation enzymes like peroxidases or polyphenol oxides leads to toxic metabolites that impede insect growth physiology and development^67-65^.

Generally, plant growth relies on the absorption and utilization of minerals and nutrients from the soils^68,69^ through extra radical mycelium networks, which act as a transmission channel of an entomopathogenic fungus to help plants to absorb nutrients and water^70^. *B. bassiana* is characterized by its capacity to enter the tomato tissue with random inoculation without adversely affecting plant growth^69^. The application of *B. bassiana* to tomato plants resulted in the heightened activity of specific defense genes, particularly those involved in the synthesis of protective compounds. Moreover, when these plants were exposed to both the fungus and the insect *B. tabaci*, there was a significant increase in the activation of defense genes, thereby strengthening the plants’ resistance to pests. This shows that *B. bassiana* could act as a natural solution for protecting tomatoes against pest invasions, thereby reducing reliance on chemical pesticides^71^.

The results of the present study showed a significant increase in ascorbic acid levels in the *B. bassiana*- treated ripe tomato fruits (red) compared to tomato fruits in the only- fertilization treatment and control; this result is in agreement with^37^**,** who found that the content of ascorbic acid in the ripe tomatoes cultivated in the field has a higher content of ascorbic acid.

Mainly, the content of ascorbic acid differentiated in accordance with some parameters such as growth, fruit maturation processes, development, phase, and the adverse conditions produced by the biotic and abiotic stress factors, climatic changes, especially temperature, and environmental elements, especially the exposures to the dark and the light^37,72^.

The driving force behind vitamin C importance to humans is that it cannot be synthesized by them, so they should get it only from superior plants that are able to synthesize that type of vitamin^40^. A dosage of 10 mg/day for a person prevents avitaminosis C, but the recommended amount for an adult is about 60 mg/day^73^.

## Conclusion

*Beauveria bassiana*, an entomopathogenic fungus, presents a promising natural biocontrol option for *Bemisia tabaci*, a major pest impacting crops like tomatoes, serving as an effective alternative to traditional chemical pesticides. Utilizing *B. bassiana* not only as a biopesticide but also as a growth enhancer aligns with the growing demand for sustainable, pesticide-free agricultural practices. The multifunctional properties of entomopathogenic fungi extend beyond pest control, offering benefits such as improved plant health and enhanced nutritional quality of crops. Incorporating EPFs into agricultural practices can synergistically enhance plant defenses against biotic threats such as pests and promote growth under abiotic stress conditions. *B. bassiana* is a vital tool for modern agriculture, providing an integrated solution to pest management and plant health due to its dual purpose of growth enhancement and pest control. Overall, the integration of *Beauveria bassiana* within farming systems represents a significant advancement towards fostering environmentally sustainable agricultural practices that meet the challenges of global food security while preserving ecological integrity.

## Data Availability

The datasets used and/or analyzed during the current study are available from the corresponding author on reasonable request.
